# Supplementary material for: Acute kidney disease stage predicts outcome of patients on extracorporeal membrane oxygenation support
Source: PLoS One. 2020 Apr 8;15(4):e0231505. doi: 10.1371/journal.pone.0231505 (PMC7141841; doi:10.1371/journal.pone.0231505)
Supplement: Attachment — Submitted filename: Response to Reviewers.docx [file pone.0231505.s002.docx]

- **Title:** Acute Kidney Disease Stage Predicts Outcome of Patients on Extracorporeal Membrane Oxygenation Support

**Authors:** Cheng-Kai Hsu, I-Wen Wu, Yih-Ting Chen, Tsung-Yu Tsai, Feng-Chun Tsai, Ji-Tseng Fang, Yung-Chang Chen

**Manuscript No.:** PONE-D-19-31910

**Index of Changes made in the revised manuscript (highlighted version):**

**Page 1, line 1:** Revised the title “Acute Kidney Disease Stage Predicts Outcome of Patients on Extracorporeal Membrane Oxygenation Support”.

**Page 2, line 30:** Added median survival time of patients with stage 0.

**Page 4, line 51:** Added “The global burden of AKI is around 13.3 million cases per year with hospitalizations for AKI rising over time. A published meta-analysis showed that in the United States alone, AKI is associated with 1 hospitalization every 7.5 minutes”.

**Page 4, line 55:** Added “A recent meta-analysis study also reported that the incidence rate of AKI in patients receiving ECMO is as high as 62.8%, and that in-hospital mortality is 3.7 times higher in patients on ECMO who develop AKI that require renal replacement therapy. Another previously published meta-analysis showed that the incidence of AKI and severe AKI in pediatric patients who require ECMO are high.”.

**Page 5, line 72:** Added definition of AKD.

**Page 8, line 116:** Added “We determined the estimated glomerular filtration (eGFR) using the CKD epidemiology collaboration equation.”.

**Page 9, line 127:** Added “Baseline serum creatinine level was defined as the lowest serum creatinine level in the 30 days preceding ECMO support or the lowest serum creatinine level in the 30 days following ECMO support if serum creatinine levels were not measured in the 30 days before ECMO support”.

**Page 11, line 154:** Added “, and they consisted of 120 patients without CKD at baseline and 48 patients with CKD at baseline (stage 3: N=36, stage 4: N=7, stage 5: N=5). There were 93 patients without AKD and 75 patients with AKD (Fig. 2). Of the 75 patients with AKD, 30, 13, and 32 patients had stage 1, stage 2, and stage 3 AKD, respectively.”.

**Page 13, line 176**: Added “the types of ECMO (veno-arterial or veno-venous)”.

**Page 14, line 195**: Added “baseline eGFR”.

**Page 16, line 212**: Added “baseline eGFR”

**Page 19, line 250**: Added “with relatively low renal reserves, proteinuria leading to loss of nephron mass and hyperfiltration”

**Page 20, line 264:** Added “Fourth, other variables such as quantity of intravenous fluid, volume balance, transfusion, intravenous contrast medium use, baseline medication, and baseline comorbidities were not measured in our study.”

**Table 1:** Added eGFR values

**Table 2:** Added “types of ECMO” variable.

**Table 3:** Added eGFR values

**Table 4**: Added eGFR values and AKI variables

**Figure 2:** Totally revised.

- **Title:** Acute Kidney Disease Stage Predicts Outcome of Patients on Extracorporeal Membrane Oxygenation Support

**Authors:** Cheng-Kai Hsu, I-Wen Wu, Yih-Ting Chen, Tsung-Yu Tsai, Feng-Chun Tsai, Ji-Tseng Fang, Yung-Chang Chen

**Manuscript No.:** PONE-D-19-31910

**Answers to Editor:**

1. The editor asked for the information about the types of ECMO. Among 168 patients, 116 patients received veno-arterial ECMO and 52 patients received veno-venous ECMO. We revised the Table 2 and added the description in the text. (Table 2; page 13, line 176-177, 179-180)

2. The editor asked for the information about the duration of ECMO support and the differences between subgroups of patients. The median duration of ECMO support of all patients was 7 (IQR: 4-12) days, and AKD subgroup had longer duration of ECMO support (9 versus 6 days, *p*=0.024) than non-AKD subgroup. AKD stage was still significant independent predictor for survival after adjusting for the duration of ECMO support. (Table 1; page 11, line 160-162; Table 3; page 14, line 194-195).

3. The editor suggested describing a clear sequential story of renal failure in the form of a flowchart. We modified Figure 2 according to editor’s valuable advice. The incidence of AKI was 78.8%. Among 168 patients, 58 patients developed AKI stage 3 and 56 patients received CRRT support with a median duration of 8 days. Among 122 patients with AKI, 62 patients developed AKD. Nineteen patients developed CKD 90 days later. Among 122 patients with AKI, 40 patients did not progress to CKD 90 days later. (Figure 2)

4. The editor asked for language editing. We are very sorry for our poor English. The manuscript had received English editing according to Editor’s suggestion.

**Answers to Reviewer 1:**

1. The reviewer proposed concern on the focus of relative lower incidence of AKD/AKI. We modified Figure 2, and showed that the incidence of AKI was 78.8% among 260 ECMO patients. Among patients who survived more than 7 days, the incidence of AKD was 44.6%. (Figure 2)

2. The reviewer asked for the information about the number of patients who developed stage 3AKI and AKD. Among 75 patients with AKD, 30, 13, and 32 patients were stage 1, stage 2, and stage 3 AKD respectively. Among 122 patient with AKI, 58 patients developed severe AKI stage 3. We added the information to the Figure 2 and the text. (Figure 2; page 11, line 154-158)

3. The reviewer provided important recent study about the ECMO patients who develop AKI requiring RRT carry 3.7-fold higher hospital mortality (PMID:31284451). We added this reference and modified the introduction section. (page 4, line 55-58)

4. The reviewer asked for the information about AKI recovery. We modified Figure 2 and described a more clear sequential story of renal failure in the form of a flowchart. Among 122 AKI patients, 60 patients did not develop AKD and 40 patients did not develop CKD. Among 56 severe AKI patients who received CRRT and survived more than 90 days, 3 patients were able to be off dialysis. (Figure 2)

5. The reviewer asked for the information about indication for ECMO and types of ECMO (VA or VA modes). We revised the Table 2 and added the description in the text. The indication and diagnosis of patients needing ECMO support were shown in Table 2. Among 168 patients, 116 patients received veno-arterial ECMO and 52 patients received veno-venous ECMO. (Table 2, page 13, line 176-177, 179-180)

**Answers to Reviewer 2:**

1.The review recommended for additional editing for English grammar throughout the manuscript. The manuscript had received English editing according to Editor’s suggestion.

2. The reviewer provided information about recent meta-analysis (PMID 31284451). We added this important reference and modified the introduction section. (page 4, line 50-52)

3. The reviewer proposed concerns on whether the study is prospective or retrospective. Our study was retrospective cohort study, and we had modified our methods section and Figure 2 flowchart. (page 4, line 55-58)

4. The reviewer asked for information about types of ECMO. Among 168 patients, 116 patients received veno-arterial ECMO and 52 patients received veno-venous ECMO. We revised the Table 2 and added the description in the text. (Table 2; page 13, line 176-177, 170-180)

5. The reviewer asked for the information about the duration of ECMO support and the differences between subgroups of patients. The median duration of ECMO support of all patients was 7 (IQR: 4-12) days, and AKD subgroup had longer duration of ECMO support (9 versus 6 days, *p*=0.024) than non-AKD subgroup. AKD stage was still significant independent predictor for survival after adjusting for duration of ECMO support. (Table 1, page 11, line 160-162; Table 3, page 14, line 194-195).

6. The reviewer proposed concern on whether we recruited only patients with ECMO support having concomitant AKI. We are so sorry about the written mistake in methods section. Actually, we enrolled patients under ECMO support with AKI and without AKI. We modified manuscript and Figure 2 to describe a more clearly sequential story of renal failure in the form of a flowchart. (Figure 2; page 7, line 96-98; page 11, line 154-158)

7. The reviewer proposed concern on “the highest serum creatinine value for staging AKI and AKD”. We adjusted Figure 2 and text to clarify the issue. We enrolled patients with ECMO support who survived more than 7 days with and without AKI. The index date was the first date of ECMO support. The highest serum creatinine value within 7 days was used for staging AKI and 7-90 days for staging AKD. (Figure 2; page 8, line 121-122)

8. The reviewer asked for the detail about how was baseline creatinine determined. Baseline serum creatinine was defined as the lowest serum creatinine level in the 30 days preceding ECMP support or the lowest serum creatinine level in the 30 days following ECMO support if serum creatinine levels were not measured in the 30 days before ECMO support. (page 9 127-130)

9. The reviewer suggested that other variables would be useful to include in this report. We reviewed our data and the prevalence of baseline CKD was 28.6%. We added the information in the text. Other variable such as quantity of fluid, volume balance, and baseline medication were not available in our data source. We added these factors into our limitation section. (page 11, line 154-158; page 20, line 264-267)

10. The reviewer asked for the information of median survival time for AKD stage 0 (non-AKD) patients. More than half of the non-AKD patients survived 10 years after ECMO support, so the median survival time for non-AKD patients was more than 10 years. We added this information in the text. (page 14, line 186-187)

11. The reviewer proposed concern on whether urine output, diuretics use, and volume balance influenced outcome. The urine output on day 1 of ECMO was an independent predictor for survival in our study. AKD stages were also independent predictors for survival after adjusting or not adjusting urine output on day 1 of ECMO. Volume status and diuretics use were not available in our data source. This is the limitation of our study. We added these in the text. (page 20, line 264-267)

12. The reviewer proposed concern on the impact of urine output or AKI on subsequent AKD. The reviewer also suggested using the eGFR (CKD-EPI equation) for confounder adjustment. Our data demonstrated urine output on day 1 of ECMO support was a significant independent risk factor of AKD after adjusting other variables. However, AKI was not an independent risk factor of subsequent AKD. We replaced serum creatinine value with eGFR (CKD-EPI equation) according to reviewer’s opinion. We modified Table 1, Table 3, Table 4, and the text. (Table 1; Table 3; Table 4; page 11, line 165-166; page 14, line 194-195; page 16, line 212)

13. The reviewer asked for whether the presence of AKD is related to increase in CKD, which thus translated to worse survival and would a patient who developed AKD that resolved without developing CKD possess the same worse outcomes. We modified Figure 2 to describe a sequential story of renal failure in the form of a flowchart. Seventy-seven patients died within 7-90 after ECMO support and 5 patients died 90 days after ECMO support. Among the patients who survived more than 90 days, only 19 patients developed CKD. Consequently, the majority of mortality could not be attribute to the complication of CKD. The patients who developed AKD that resolved without developing CKD seemed to have better outcome than patients with CKD. Among AKD patients who survived more than 90 days, 17 patients did not developed CKD and the mortality rate was 29.4%; 12 patients developed CKD and the mortality rate was 58.3%. However, the case number was too small to be further analyzed. (Figure 2)

14. The reviewer suggested stratified ECMO patients by primary diagnosis. However, this is a singer center study with a relatively small case number. We had described this limitation in the text. (page 20, line 267-271)

15. The reviewer asked for the information of patients requiring RRT. Among 58 stage 3 AKI patients, 56 patients had received CRRT. (Figure 2)

16. The reviewer proposed concern on how pre-existing CKD cause progression of AKI to CKD. Patients with CKD have relatively small renal reserves, and they are vulnerable to develop renal progression after AKI insult which may be attributed to further increase in single nephron glomerular pressure. (page 19, line 250-251)

17. The reviewer proposed concern on exclusion of patients who survived less than 7 days. Among initial 260 ECMO patients, 92 patients died within 7 days. We must exclude these patients because the diagnosis of AKD is based on the renal function of 7-90 days after renal injury. (Table 2)

18. The reviewer asked for the information of amelioration of AKD severity. We think it would be similar to management with patients with CKD but we can intervene in earlier period (7-90 days after renal injury). Renin-angiotensin system blockers, sodium-glucose cotransporter 2 inhibitors, and anti-inflammatory agents may play a role.

19. The reviewer asked for the information about AKI biomarkers. Novel AKI markers such as NGAL were not measured in our study. So this may have led to underestimation of patients with stage 0 as we categorized patients with AKD stage 0 as the non-AKD group. (page 19, line 260-262)

20. The reviewer asked for the different diagnosis effects of AKI, AKD and CKD with survival in ECMO patients. Our data demonstrated that AKI stage was independent predictors for 30-day survival and AKD was independent predictors for 10-year survival. However, only 19 patients who survived more than 90 days developed CKD. Consequently, our data is too small to analyze the effect of CKD on outcome. AKI and AKD may be used as short-term and long-term predictors, respectively. (Figure 2)

**Answers to Reviewer 3:**

1.The reviewer proposed concern on the inclusion criteria. We are so sorry about the written mistake in methods section. Actually, we enrolled patients under ECMO support with AKI and without AKI. We modified manuscript and Figure 2 to describe a more clearly sequential story of renal failure in the form of a flowchart. (Figure 2; page 7, line 96-98)

2. The reviewer asked for the time frame for assessment of AKD. The index date was the day 1 of ECMO support. AKD was defined 7-90 days after ECMO support. We modified Figure 2 to demonstrate the time frame for assessment of AKD. (Figure 2)

3. The reviewer suggested deleted “10-year cohort study” in this manuscript. We adjusted our title and manuscript according to reviewer’s opinion. (page 1, line 1-2)

4. The reviewer asked for the detail about how was baseline creatinine determined. Baseline serum creatiine level was defined as the lowest serum creatinine level in the 30 days preceding ECMO support or the lowest serum creatinine level in the 30 days following ECMO support if serum creatinine levels were not measured in the 30 days before ECMO support. (page , line 127-130)

5. The reviewer provided important recent study about the ECMO patients who develop AKI requiring RRT carry 3.7-fold higher hospital mortality (PMID:31284451). We added this reference and modified the introduction section. (page 4, line 55-58)

6. The reviewer asked for how did we obtain mortality outcome after hospital discharge. We linked patients’ ID to Taiwan’s National Health Insurance Research Database (NHIRD) to obtain the date of mortality.

7. The reviewer asked for the information of adjusting covariates selection. We selected basic demographic covariates including age, gender, baseline eGFR and other covariates that had *p* value > 0.2 for univariate analysis between two groups.

8. The reviewer proposed concern on index date for survival analysis. We defined index date as the day 1 of ECMO support. (Figure 2)

9. The reviewer asked for language editing. We are very sorry for our poor English. The manuscript had received English editing according to reviewer’s opinion.

**Answers to Reviewer 4:**

1. The reviewer suggested describing a whole story of AKI to CKD. We modified Figure 2 according to reviewer’s valuable advice. The incidence of AKI was 78.8%. Among 168 patients, 58 patients developed AKI stage 3 and 56 patients received CRRT support with a median duration of 8 days. Among 122 patients with AKI, 62 patients developed AKD. Nineteen patients developed CKD 90 days later. Among 122 patients with AKI, 40 patients did not progress to CKD 90 days later. (Figure 2)

2. The reviewer asked for describing the incidence of AKI in the first seven days and its impact on outcomes including mortality. In our study, 205 patients developed AKI with incidence rate of 78.5%. The impact of AKI on mortality did not be mentioned in our manuscript because we excluded the patients who died within 7 days after ECMO support (N=92). We revised Figure 2 to describe a sequential story of renal failure and the number of exclusion in the form of a flowchart. (Figure 2)

3. The reviewer asked for evaluating the duration of renal injury. There were 205 patients with AKI (0-7 days), 75 patients with AKD (7-90 days), and 19 patients with CKD (>90 days). We revised Figure 2 to describe the detail of flowchart. (Figure 2)

4. The reviewer suggested using a flow diagram to demonstrate that how many patients with AKI developed AKD later. Among patients with AKI who survived more than 7 days after ECMO support (N=122), 62 patients developed AKD in the period of 7-90 days after ECMO support. We revised the Figure 2 according to reviewer’s valuable suggestion.

5. The reviewer asked for the number of patients who developed CKD. A total of 19 patients developed CKD 90 days after ECMO support. (Figure 2)

6. The reviewer asked for the information of CRRT number and duration, and the practice for CRRT on ECMO. Among patients with AKI stage 3 who survived more than 7 days after ECMO support, 56 patients received CRRT. All patients received continuous veno-venous hemofiltration (CVVH). We separated vascular access and CRRT machine from ECMO circuit. Accordingly, additional vascular access points must be used. The method provided the advantage of no interference with ECMO hemodynamic while having the ultrafiltration controlled by the CRRT machine.

7. The reviewer asked for the information of mortality in the whole cohort. Among 168 patients with ECMO support who survived more than 7 days, 82 patients expired in the 10-year follow-up. The mortality rate was high and we used many variables in the regression analysis for adjustment.

8. The reviewer suggested shortening the results section. We revised out results section according to reviewer’s opinion. (page 11, line 160-166)

9. The reviewer asked for the information of tests of normality. The Kolmogorov-Simirnov method was used to test normality of numerical variables. Differences in continuous variables between the AKD group and non-AKD group were analyzed by Student t-test or Mann Whitney U test according to results of test of normality. (page 9 line 142-143)

**Answers to Reviewer 5:**

1. The reviewer suggested describing epidemiology on ECMO and AKI and providing the definitions of the AKD in the introduction section. A recent meta-analysis study reveals that among patients receiving ECMO, the incident rate of AKI is 62.8%. The AKD stage 1 was defined as an increase of serum creatinine to 1.5-1.9 times, stage 2 as an increase of serum creatinine to 2.0-2.9 times, and stage 3 as an increase of serum creatinine ≥ 3.0 times, from the baselines in the period of 7-90 days after renal injury. We revised the introduction section according to reviewer’s suggestion. (page 4, line 56-58; page 5, line 72-75)

2. The reviewer provided important recent study about the ECMO patients who develop AKI (PMID:31284451). We added this reference and revised the introduction section. (page 5, line 72-75)

3. The reviewer suggested describing more data in the patient schema. We modified Figure 2 according to reviewer’s valuable advice. The incidence of AKI was 78.8%. Among 168 patients, 58 patients developed AKI stage 3 and 56 patients received CRRT support with a median duration of 8 days. Among 122 patients with AKI, 62 patients developed AKD. Nineteen patients developed CKD 90 days later. (Figure 2)

4. The reviewer asked for the detail about how was baseline creatinine determined. Baseline serum creatinine level was defined as the lowest serum creatinine level in the 30 days preceding ECMP support or the lowest serum creatinine level in the 30 days following ECMO support if serum creatinine levels were not measured in the 30 days before ECMO support. (page 9, line 127-130)

5. The reviewer asked for the information about admission serum Creatinine between AKD and non-AKD groups. The mean serum creatinine on first day of ECMO support were 2.2 and 2.5 mg/dL in non-AKD and AKD group, respectively. There was no statistically significant difference (*P* = 0.208).

6. The reviewer asked for the information about medications which could potentially influence the serum creatinine. Other variable such as quantity of fluid, volume balance, and baseline medications were not available in our data source. We added these factors in the limitation section. (page 20, line 264-267)

7. The reviewer asked for information about types of ECMO. Among 168 patients, 116 patients received veno-arterial ECMO and 52 patients received veno-venous ECMO. We revised the Table 2 and added the description in the text. (Table 2; page 13, line 176-177, 179-189)

8. The reviewer proposed concern on AKD staging for risk stratification in this critically ill patient. We focused on the impact of AKD on survival, so we must exclude the patient who expired within 7 days since ECMO support. We can consider intervening in this earlier period (7-90 days after insult of renal injury) via renin-angiotensin system blockers, fluid balance management, avoidance of nephrotoxic agents. Further novel agents such as sodium-glucose cotransporter 2 inhibitors and anti-inflammatory agents may also play a role.

**Answers to Reviewer 6:**

1. The reviewer provided important recent study about the pediatric patients with ECMO support who develop AKI . We added “Another previously published meta-analysis showed that incidences of AKI and severe AKI in pediatric patients who require ECMO are high.” in the text. (page 4, line 58, page 5, line 59)

2. We deleted the sentence “The AKD is an emerging medical entity in the field of critical care and the clinical significance of this kidney condition remains unraveled to date.” according to reviewer’s suggestion.

3. We are so sorry about the spelling mistake of KDIGO. We have revised the manuscript according to reviewer’s opinion.

4. The reviewer suggested citing a reference about the burden of AKI. We added ”The global burden of AKI is around 13.3 million cases per year with hospitalizations for AKI rising over time. A published meta-analysis showed that in the United States alone, AKI is associated with 1 hospitalization every 7.5 minutes. ” in the introduction section according to reviewer’s suggestion. (page 4, line 51-53)

5. The reviewer proposed concerns on the definition of AKI and AKD. We defined AKI according to KDIGO criteria in which renal failure developed within 7 days after ECMO support. AKD was defined as the clinical condition in which renal injury is present between 7-90 days after initiation of ECMO support. We modified Figure 2 and described a sequential flowchart about study process. (Figure 2; page 8, line 118-120)

6. The reviewer proposed concern on why AKI is not an independent predictor of AKD in our study. Among 92 patients who died within 7 days after ECMO support, 83 patients developed AKI. We must exclude the patient who died within 7 days because AKD was defined as renal injury within 7-90 after insult. A large portion of patients with AKI who died within 7 days after ECMO support may explain why AKI is not an independent predictor of AKD in our result.

7. Our manuscript had received English language editing according to reviewer’s suggestion.
