# Supplementary material for: Acute kidney disease stage predicts outcome of patients on extracorporeal membrane oxygenation support
Source: PLoS One. 2020 Apr 8;15(4):e0231505. doi: 10.1371/journal.pone.0231505 (PMC7141841; doi:10.1371/journal.pone.0231505)
Supplement: Attachment — Submitted filename: Response to Reviewers.docx [file pone.0231505.s003.docx]

- **Title:** Acute Kidney Disease Stage Predicts Outcome of Patients on Extracorporeal Membrane Oxygenation Support

**Authors:** Cheng-Kai Hsu, I-Wen Wu, Yih-Ting Chen, Tsung-Yu Tsai, Feng-Chun Tsai, Ji-Tseng Fang, Yung-Chang Chen

**Manuscript No.:** PONE-D-19-31910R1

**Index of Changes made in the revised manuscript (highlighted version):**

**Page 5, line 71:** Added “occurring or persisting”.

**Page 5, line 75:** Added “that occurred or persisted”.

**Page 8, line 121:** Added “increase of serum creatinine that occurred or persisted 7 and 90 days”.

**Page 14, line 188:** Modified “16 days.”

**Page 18, line 231:** Modified “In this retrospective cohort study, we showed that stage of AKD is an independent risk factor of mortality in patients receiving ECMO support”.

**Page 19, line 247:** Added “AKI is an independent risk factor of CKD. In our study, univariate analysis showed that AKI is a significant risk factor (HR: 2.623, *p* = 0.001) of AKD among patients receiving ECMO support. However, there is no statistical significance in multivariate analysis after adjusting for age, sex, duration of ECMO support, APACHE II score, SOFA score, GCS score, MAP, urine output, serum potassium level, and baseline eGFR (HR: 2.257, *p* = 0.133). The reason may be attributed to the relative small size of our study”.

**Page 19, line 259:** Added “The intervention of ameliorating AKD severity may be similar to management with patients with CKD. Renin-angiotensin system blockers, sodium-glucose cotransporter 2 inhibitors, and anti-inflammatory agents may play a role. Further studies are necessary to investigate the effects of increasing AKD severity on outcomes of patients on ECMO and the management of amelioration of AKD severity.”

**Page 20, line 273:** Added “duration of renal replacement therapy”.

**Page 20, line 276**: Added “Fifth, other insults that cause renal injury may have occurred in the 7-90 days. This is a potential limitation not only in our study, but also in other retrospective articles regarding AKI/AKD.”.

**Page 21, line 288**: Revised “AKD staging may help in risk stratification of critically ill patients on ECMO support.”.

- **Title:** Acute Kidney Disease Stage Predicts Outcome of Patients on Extracorporeal Membrane Oxygenation Support

**Authors:** Cheng-Kai Hsu, I-Wen Wu, Yih-Ting Chen, Tsung-Yu Tsai, Feng-Chun Tsai, Ji-Tseng Fang, Yung-Chang Chen

**Manuscript No.:** PONE-D-19-31910R1

**Answers to Reviewer 2:**

1. The reviewer suggested rewriting the definition of AKD. We revised the definition of AKD according to reviewer’s suggestion. (page 5, line 71, 75; page 8, line 121)

2. The reviewer suggested adding the limitation about other insults that cause renal injury occurring in the 7-90 days. We revised the section of discussion according to reviewer’s suggestion. (page 20, line 275-277)

3. The reviewer asked for the information about baseline serum creatinine. 42 patients had a baseline creatinine that was defined in the 30 days following ECMO support. (20 patients with ARDS, 5 patients with AMI, 7 patients with myocarditis, 1 patient with decompensated heart failure, 2 patients with shock status simultaneous with hypoxic respiratory failure, 3 patients with sudden collapse, 4 patients with severe traumatic injury). We choosed lowest creatinine to use as the baseline creatinine following ECMO rather than after surgery because only 79 patients who had received surgery in our study. (72 patients with post-cardiotomy cardiogenic shock, 4 patients with severe traumatic injury, 3 patients with post-lung or heart transplantation).

4. The reviewer suggested focusing more on the association of AKD with mortality but not urine output. We revised the manuscript according to reviewer’s opinion. (page 18, line 231-232)

5. The reviewer did not see the revised Figure 2. We have re-uploaded the file of Figure 2.

6. The reviewer suggested replacing “half a month” with actual days. We revised the manuscript. (page 14, line 188).

7. The reviewer asked for the information about the mean survival time of non-AKD patients. The mean survival time of non-AKD patients was 88.5 months.

8. The reviewer proposed concern that why AKI is not a risk factor of AKD in our study. We added “AKI is an independent risk factor of CKD. In our study, univariate analysis showed that AKI is a significant risk factor (HR: 2.623, *p* = 0.001) of AKD among patients receiving ECMO support. However, there is no statistical significance in multivariate analysis after adjusting for age, sex, duration of ECMO support, APACHE II score, SOFA score, GCS score, MAP, urine output, serum potassium level, and baseline eGFR (HR: 2.257, *p* = 0.133). The reason may be attributed to the relative small size of our study.” in the section of discussion. (page 19, line 247-252)

9. The reviewer asked for the information of duration of CRRT. The information was not available in this retrospective study. We added the limitation in the section of discussion. (page 20, line 273)

10. The reviewer suggested to add hypothesis of how interventions for AKD in the text and delete “mandatory” in the text. We added “The intervention of ameliorating AKD severity may be similar to management with patients with CKD. Renin-angiotensin system blockers, sodium-glucose cotransporter 2 inhibitor, and anti-inflammatory agents may play a role. Further studies are necessary to investigate the effects of increasing AKD severity on outcomes of patients on ECMO and the management of amelioration of AKD severity” in the section of discussion. We also softened the word “mandatory” according to reviewer’s suggestion. (page 19, line 259-263; page 21, line 287-288)
